# HbA1C Variability and the Risk of Renal Status Progression in Diabetes Mellitus: A Meta-Analysis

**DOI:** 10.1371/journal.pone.0115509

**Published:** 2014-12-18

**Authors:** Dongsheng Cheng, Yang Fei, Yumei Liu, Junhui Li, Qin Xue, Xiaoxia Wang, Niansong Wang

**Affiliations:** Department of Nephrology and Rheumatology, Shanghai Jiaotong University Affiliated Sixth People's Hospital, Shanghai, P.R. China, 200233; Baker IDI Heart and Diabetes Institute, Australia

## Abstract

**Objective:**

To explore the association between glycated hemoglobin (A1C) variability and renal disease progression in patients with diabetes mellitus.

**Methods:**

A comprehensive search was performed using the PubMed and Embase databases (up to April 26, 2014). The hazard ratio (HR) was pooled per unit increase in the standard deviation of A1C (A1C-SD) to evaluate the dose-response relationship between A1C-SD and the risk of nephropathy.

**Results:**

Eight studies with a total of 17,758 subjects provided the HR for A1C-SD and were included in the final meta-analysis. The pooled HR results demonstrated that A1C-SD was significantly associated with the progression of renal status (HR for both T1DM and T2DM 1.43, 95% confidence interval [CI] 1.24–1.64; HR for T1DM 1.70, 95%CI 1.41–2.05; HR for T2DM 1.20, 95%CI 1.12–1.28). A1C-SD was significantly correlated with new-onset microalbuminuria (HR for T1DM 1.63, 95%CI 1.28–2.07; HR for T2DM 1.23, 95%CI 1.08–1.39). These outcomes were also supported in subgroup analyses. Furthermore, sensitivity analyses demonstrated that the results were robust.

**Conclusions:**

A1C variability is independently associated with the development of microalbuminuria and the progression of renal status in both type 1 and 2 diabetes patients. A standard method for measuring A1C variability is essential for further and deeper analyses. In addition, future studies should assess the effect of reducing A1C variability on nephropathy complication.

## Introduction

Long-term glycemic stability protects against vascular complications in both type 1 [1] and type 2 diabetes mellitus [2]. Abundant evidence and guidelines have defined appropriate A1C levels, a marker of mean blood glucose levels, as a priority therapeutically [3].

Evidence has suggested that chronic hyperglycemia is responsible for the development of complications in diabetes patients. In recent years, a number of studies have focused on whether glucose variability could be a predictor of complications. However, glucose variability could be defined in several ways: within-day variability, between-day variability, and long-term variability expressed using changes in A1C [4]. A post-hoc analysis of the Diabetes Control and Complications Trial (DCCT) first described that A1C variability, similar to mean A1C levels, could predict the development of nephropathy and retinopathy in T1DM patients [5]. Another analysis performed by Sugawara et al [6] also demonstrated that the intra-person standard deviation in A1C (A1C-SD) was an independent risk factor for the development of microalbuminuria in T2DM. Recently, an increasing number of studies have focused on the correlation between A1C variability and the risk of nephropathy. However, no systemic review has been performed on this subject. Therefore, we conducted this meta-analysis to evaluate the effect of A1C variability on nephropathy complications in patients with diabetes mellitus.

## Methods

This review was conducted and reported according to PRISMA (Preferred Reporting Items for Systematic Reviews and Meta-Analysis; S1 Checklist).

### Search strategy

A literature search was performed using the PubMed and Embase databases with no language restrictions to identify studies published before April 26, 2014. The main search term was a combination of MESH terms and text words for glycated hemoglobin variability and nephropathy. The detailed search strategies are presented in S1 File. The reference lists of all identified studies were also checked to identify any additional relevant studies. All literature management was performed using Endnote X4.

### Study selection criteria

The study selection criteria were as follows. (1) Studies that measured A1C variability, either as the coefficient of variation of A1C (A1C-CV) or A1C-SD. (2) Studies that investigated the association between A1C variability and the progression of renal status (defined as microalbuminuria development, any increase in albuminuria, or the progression to chronic kidney disease, which was defined as an estimated glomerular filtration rate (eGFR) <60 ml/min/1.73 m^2^ or the progression to ESRD). (3) Hazard ratios (HRs) and their 95% confidence intervals (CI) could be extracted.

### Data extraction

DC and FY extracted the data independently using electronic extraction forms. The extracted data included the authors, study titles, publication year, country, age of patients at enrollment, number of subjects, study design, follow-up duration, the definition of A1C variability, HR and 95%CIs, variable adjustment, and renal outcome. Discrepancies were resolved by consensus or by consultation with a third reviewer.

### Quality assessment

A nine-score system of the Newcastle–Ottawa quality assessment scale (NOS) was applied to assess the quality of the included studies according to three broad perspectives: the selection of the study groups (0–4 points), the comparability of the groups (0–2 points), and the ascertainment of either the exposure or outcome of interest (0–3 points) [7]. Disagreement on the score was resolved by discussion between reviewers.

### Data analysis and synthesis

The HRs were pooled per unit increase in A1C-SD to estimate the dose-response associations between A1C-SD and the risk of nephropathy. A1C-SD was either reported in the included publications or was calculated from the categorical data by estimating the generalized least-squares trend [8]. A random-effects model was applied for the pooled analyses. For studies that performed classifications using quartiles of standard deviations [9], the midpoints of the lower and upper quartiles were assigned to the HR of the corresponding category. For cases in which the lowest or highest category was open-ended, the length of the category was estimated using the amplitude of the adjacent ones. The pooled HR for A1C-CV was absent from the final meta-analysis because of a lack of sufficient trials. The heterogeneity across trials was assessed using χ^2^ tests (*P* <0.10) and *I^2^* tests. *I^2^* values of 25%, 50%, and 75% corresponded to low, medium, and high levels of heterogeneity, respectively [10]. Subgroup analyses were performed based on the types of diabetes mellitus, age (adolescent or adult), renal outcome (microalbuminuria onset, the exacerbation of renal function or albuminuria), and sample size (<1000 or ≥1000). Sensitivity analyses were conducted by eliminating unpublished trials, excluding trials with a mean follow-up duration <5 years, removing trials that reported crude SDs, and by calculating fixed effects models. Publication bias was determined using Egger's test. As reported previously [11], the sensitivity of Egger's tests is generally low when <20 trials are included; therefore, the trim and fill method was used to estimate the influence of the missing studies [12]. Data analyses were performed using STATA software (Version 12. College Station, TX, USA).

## Results

### Search results and characteristics of the included studies

**Figure 1:**
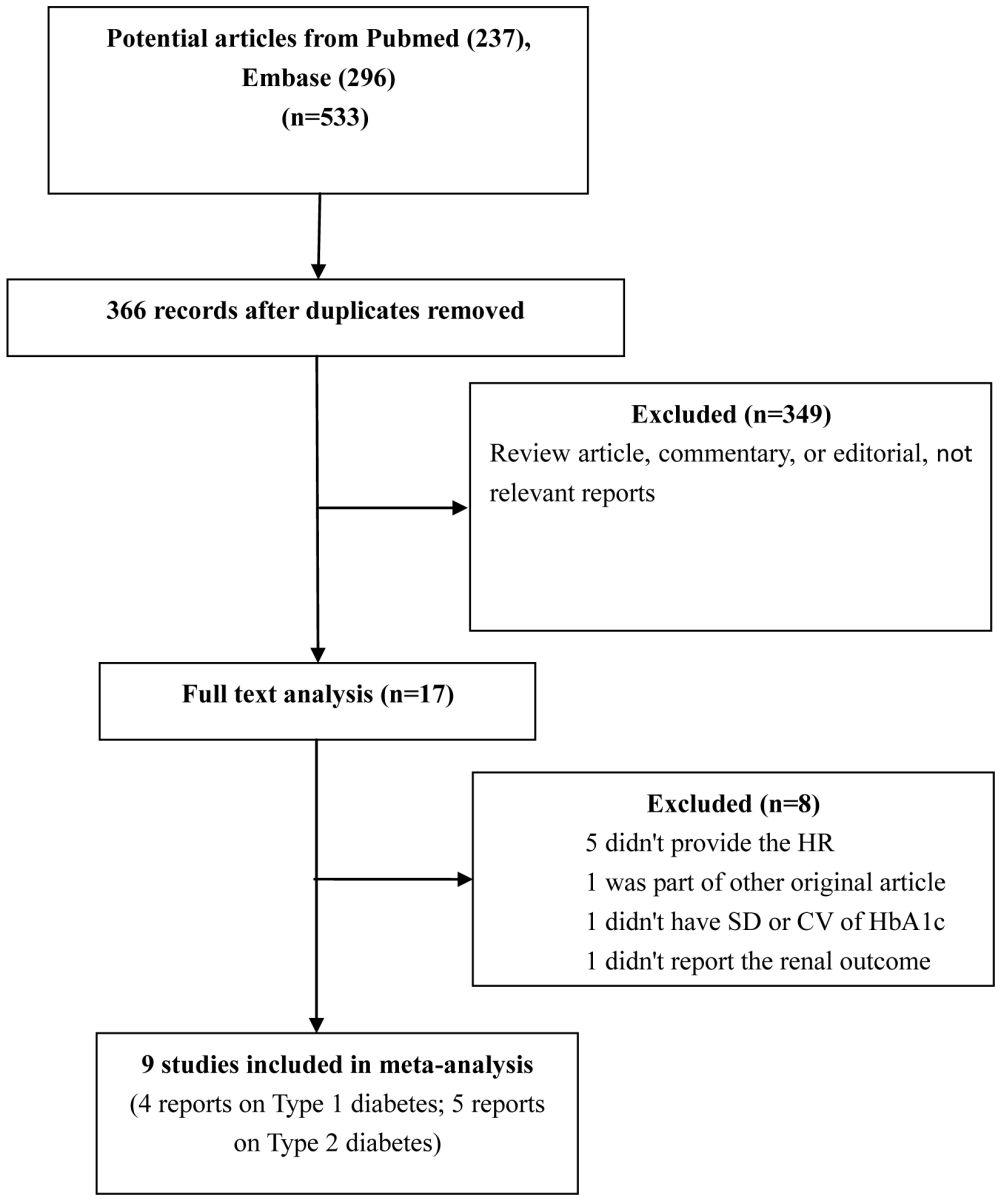
Study flow chart of trial selection and exclusion.

Fig. 1 shows a flow chart of the trial selection process. A total of 533 relevant records from electronic databases were identified, and 366 were kept after removing duplicates. The full text of 17 of these publications was reviewed after reading the title and abstract. Ultimately, nine studies including one conference abstract (four and five studies on T1DM and T2DM, respectively) were retrieved for this review [5]–[6], [9], [13]–[18]. The characteristics of the included trials (eight cohort studies and one post-hoc RCT study) are presented in Table 1. A1C variability was expressed in several ways: A1C-CV in one cohort trial, A1C-SD in seven trials, and both in one study.

**Table 1:** Characteristics of studies included in this analysis.

Trial	Number(sex)	Age at enrollment(years)	Country(follow-up time)	Design	A1C variability	Mean A1C(%)SD	Renal Outcome	HR(95% CI)	Variable Adjustment	NOSScore
**Type 1**										
Kilpatrick 2008 (DCCT)[5]	1441 (Male: 52.8%)	27 ±7*	USA Canada (6.5 years)	Post-hoc RCT	aSD	mean:8.5$ aSD:0.73	microalbuminuria development	1.80 (1.37–2.42)	age,sex, diabetes duration, randomization treatment, prevention cohort and A1Cat the eligibility stage	4/2/3
Wadén 2009 (FinnDiane)[13]	2107 (Male: 53.2%)	36.4 ±11.8	Finland (5.7 years)	Retrospective cohort	cSD	mean:8.5 cSD:0.78	any increase in albuminuria or progression to ESRD	1.92 (1.49–2.47)	sex, diabetes duration, systolic BP, lipid, ever smoking, number of A1Cmeasurements and mean A1C	4/2/3
Marcovecchio 2011[14]	1232 (Male: 55.4%)	9.2(5.7–11.7) #	UK (NR)	Retrospective cohort	aSD	mean:9.5 aSD:0.91	microalbuminuria development	1.31 (1.01–1.70)&	age at diagnosis, sex, chronologic age, mean A1C	4/2/1
Raman 2011[15]	893 (Male: 46.9%)	8.17±3.73 #	USA (7.0 years)	Retrospective cohort	cSD	NR	microalbuminuria development	1.91 (1.37–2.66)	age, sex, race and mean A1c	2/2/1
**Type 2**										
Sugawara 2012 [6]	812 (Male: 68.7%)	54.9 ±10.4	Japan (4.3 years)	Prospective cohort	aSD	mean:7.1 aSD:0.61	microalbuminuria development	1.35 (1.05–1.72)	age,sex, diabetes duration, systolic BP, BMI, lipid, smoking history and mean A1C	4/2/1
Hsu 2012[9]	821 (Male: 46.1%)	51.2±8.3#	China Taiwan (6.2 years)	Prospective cohort	aSD	mean:7.9 aSD:1.03	microalbuminuria development	1.19 (1.03–1.38)	age at diabetes onset, sex, education, diabetes duration, smoking status, waist circumference, lipid, BP and mean A1C	4/2/3
Rodríguez-Segade 2012 [16]	2013 (Male: 47.7%)	59.2±10.6	Spain (6.6 years)	Retrospective cohort	aSD	mean:7.6 aSD:0.94	development or progression of nephropathy	1.37 (1.12–1.69)	age, sex, diabetes duration, BMI, retinopathy status, drug use, smoking status, lipid, cohort, number of A1Cmeasurements, A1Cat baseline and updated mean A1C	4/2/2
					CV			1.03 (1.01–1.04)		
Luk 2013 (The Hong Kong Diabetes Registry) [17]	8439 (Male: 47%)	57.6±13.2	China Hongkong (7.2 years)	Prospective cohort	aSD	mean:7.5 aSD:0.8	Incident chronic kidney disease: eGFR <60 ml/min/1.73 m2	1.16 (1.10–1.22)	age, sex, smoking, diabetes duration, BMI, waist circumference, BP, lipid, log urine ACR, estimated GFR, haemoglobin, drug use and mean A1C	4/2/2
							ESRD	1.53 (1.35–1.73)		
Lin 2013[18]	3220 (Male: 51.4%)	57.2±10.8	China Taiwan (4.4 years)	Retrospective cohort	CV	NR	incident chronic kidney disease: eGFR<60 ml/min/1.73 m2	1.58 (1.19–2.11)	age, sex, lifestyle factors, hypertension, baseline drug use, hyperlipidemia, BMI, diabetes-related diseases, mean fasting blood glucose and mean A1C	4/2/1

* In this trial, 16% of included individuals were adolescent (13–18 years); $, A1C at the study eligibility stage; #, Age at diagnosis; &, an erratum HR from the author; aSD, adjusted SD of A1C; cSD, crude SD of A1C; CV, the coefficient of variation of A1C; NR, not reported; BP, blood pressure; BMI, body mass index; A1C, glycated hemoglobin A1C; eGFR, estimated glomerular filtration rate; ACR, albumin-creatinine ratio; HR, hazard ratio.

In the end, eight studies that reported HRs for A1C-SD for a total of 17,758 subjects were included in the final meta-analysis. These studies were performed in seven countries (Canada, China, Finland, Japan, Spain, the United Kingdom, and the United States). The mean follow-up durations were between 4.3 and 7.2 years. The enrollment sample size ranged from 812–8439 subjects.

Of these eight studies, two reported crude SDs and six provided adjusted SDs for A1C. The frequency of A1C measurements ranged from two to 20. The mean A1C ranged from 7.1% to 9.5%, whereas the mean A1C-SD was 0.59–1.03. All studies were conducted using multivariate Cox's proportional hazards models after adjustment for mean A1C or A1C at baseline. Other common adjustment variables included age, gender, blood pressure, and diabetes duration.

Four of the enrolled trials analyzed each T1DM and T2DM. In addition, five studies established an independent association between A1C-SD and the development of microalbuminuria, of which three were on T1DM and two on T2DM. The remaining three articles, one on T1DM and two on T2DM, reported the following subjects as renal endpoints: any increase in albuminuria or the progression to ESRD, incident chronic kidney disease, and the development or progression of nephropathy. The NOS scores ranged from five to nine.

### Main meta-analysis results

**Figure 2:**
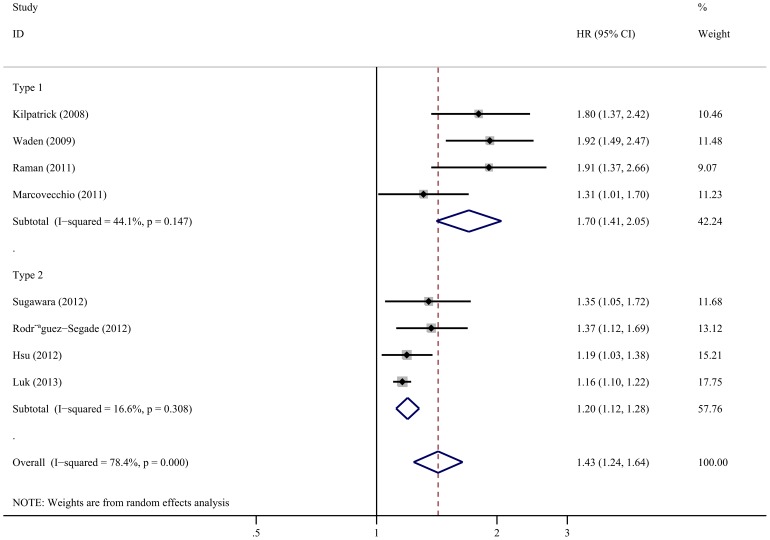
Forest plot of the relationship between A1C-SD and the progression of renal status.

Comprehensive integration and analyses of both T1DM and T2DM revealed a significant correlation between A1C-SD and the risk of renal progression (HR 1.43, 95%CI 1.24–1.64). The heterogeneity was high (*I^2^* = 78.4%; *p* = 0.000; Fig. 2). Subgroup analyses were also conducted according to the type of diabetes. Results for T1DM patients illustrated that A1C-SD was significantly associated with the progression of renal status (HR 1.70, 95%CI 1.41–2.05); the heterogeneity was moderate (*I^2^* = 44.1%; *p* = 0.147; Fig. 2). For T2DM patients, A1C variability increased the risk of progression of renal status (HR 1.20, 95%CI 1.12–1.28), and the heterogeneity was low (*I^2^* = 16.6%; *p* = 0.308; Fig. 2).

### Subgroup analysis

Subgroup analyses were performed regarding renal outcome, which indicated that A1C-SD was significantly associated with the risk of microalbuminuria onset (HR for T1DM 1.63, 95%CI 1.28–2.07; HR for T2DM 1.23, 95%CI 1.08–1.39). Other subgroup analyses were performed according to age and sample size. The association between A1C-SD and renal endpoints was consistently positive and significant among subgroups (Table 2).

**Table 2:** Subgroup analysis of the relationship between A1C-SD and renal disease.

Subgroup	Trial	Pooled HR(95% CI)	*I^2^* (*p* value)
**Type 1**			
**Age**			
Adolescent	14–15	1.56 (1.08, 2.25)	*I^2^* = 67.4% (*p* = 0.08)
Adult	5,13	1.87 (1.54, 2.25)	*I^2^* = 0% (*p* = 0.740)
**Renal outcome**			
Microalbuminuria onset	5,14–15	1.63 (1.28,2.07)	*I^2^* = 50.2% (*p* = 0.134)
Exacerbation of renal function or albuminuria	13	1.92 (1.48, 2.47)	NA
**Sample Size**			
≥1000	5,13–14	1.65 (1.30, 2.09)	*I^2^* = 58.0% (*p* = 0.093)
<1000	15	1.91 (1.37, 2.66)	NA
**Type 2**			
**Renal outcome**			
Microalbuminuria onset	6,9	1.23(1.08,1.39)	*I^2^* = 0% (*p* = 0.389)
Exacerbation of renal function or albuminuria	16–17	1.22(1.05,1.42)	*I^2^* = 57.7% (*p* = 0.124)
**Sample Size**			
≥1000	16–17	1.22(1.05,1.42)	*I^2^* = 57.7% (*p* = 0.124)
<1000	6,9	1.23(1.08,1.39)	*I^2^* = 0% (*p* = 0.389)

NA, not applicable.

### Sensitivity analysis

Sensitivity analyses were performed by eliminating unpublished trials, excluding those with a mean follow-up duration <5 years, only including the trials that used adjusted SDs, and then calculating a fixed effects model. The effect sizes were all similar in magnitude and direction to the overall estimates (Table 3). The sensitivity analyses showed that the results were robust.

**Table 3:** Sensitivity analysis of the relationship between A1C-SD and renal disease.

Type analysis	Trial	Pooled HR (95% CI)	*I^2^* (*p* value)
**Both Type 1 and Type 2**			
Fixed model analysis	5–6, 9, 13–17	1.22(1.17,1.28)	*I^2^* = 78.4% (*p* = 0.000)
Excluding unpublished trials	5–6,9, 3–14,16–17	1.38 (1.20, 1.58)	*I^2^* = 76.4% (*p* = 0.000)
Excluding crude SD trial	5–6,9,14,16–17	1.29 (1.16, 1.44)	*I^2^* = 59.5% (*p* = 0.030)
Excluding trials with follow-up <5 years	5, 9,13,15–17	1.47 (1.23, 1.75)	*I^2^* = 84.1% (*p* = 0.000)
**Type 1**			
Fixed model analysis	5,13–15	1.69 (1.47, 1.95)	*I^2^* = 44.1% (*p* = 0.147)
Excluding unpublished trial	5,13–14	1.65 (1.30, 2.09)	*I^2^* = 58.0% (*p* = 0.093)
Excluding crude SD trial	5,14	1.53 (1.12, 2.08)	*I^2^* = 61.7% (*p* = 0.106)
Excluding trials with follow-up<5 years	5,13,15	1.88 (1.59, 2.21)	*I^2^* = 0% (*p* = 0.940)
**Type 2**			
Fixed model analysis	6,9,16–17	1.18 (1.13, 1.24)	*I^2^* = 16.6% (*p* = 0.308)
Excluding trials with follow-up <5 years	9,16–17	1.18 (1.11, 1.26)	*I^2^* = 16.8% (*p* = 0.301)

### Publication bias

Egger's tests indicated there might be a publication bias for primary outcome (*P* = 0.007). Regarding the effect of the missing studies, a trim and fill analysis was conducted. After filling five studies, the results remained positive (HR 1.18, 95%CI 1.03–1.35).

## Discussion

The results of this meta-analysis indicated that A1C variability was independently associated with the development of microalbuminuria and the progression of renal status in both type 1 and 2 diabetes patients. In addition, the relationship between A1C-SD and renal endpoints was consistently positive and significant in subgroup analysis, and sensitivity analyses indicated that the results were robust.

Hyperglycemia is closely related to diabetes complications including retinopathy, nephropathy, and cardiovascular disease (CVD). As such, significant attention is paid to whether variations in glucose are a risk factor for complications in diabetes patients. Although there are several definitions of glycemic variability, it commonly refers to intra- and inter-day fluctuations in blood glucose. However, for T1DM patients, Kilpatrick et al used data from the DCCT and did not find any evidence of a relationship between glycemic variables and the development of microvascular complications using seven-point blood glucose profiles [19]–[21]. In T2DM patients, previous evidence revealed that the coefficient of variation in fasting plasma glucose and postprandial blood glucose levels were independent predictors of cardiovascular events and mortality [22]–[24]. However, no improvements in complications were found from trials of intervention in glucose variability [4].

In recent years HbA1c variability, a new parameter for long-term glycemic variability, emerged to further reflect changes in blood glucose over long periods of time, which correlates with the changes in HbA1c levels between visits. HbA1c variability was independently associated with the development of vascular complications in diabetes patients. This relationship was supported by a comprehensive analysis of HbA1c and nephropathy in the current meta-analysis. In addition, studies that were excluded from the current analysis showed similar results. Lin et al performed a retrospective cohort study of 3,220 type 2 diabetes patients with a mean follow-up duration of 4.4 years to assess the relationship between annual variation in A1C and incident diabetic nephropathy (eGFR <60 ml/min/1.73 m^2^). After multivariate adjustment, the annual A1C-CV was significantly associated with the incidence of diabetic nephropathy. The corresponding HR for the third vs. the first tertile of annual A1C-CV was 1.58 (95%CI, 1.19–2.11) [18]. An additional study was a cross-sectional analysis of the Renal Insufficiency and Cardiovascular Events (RIACE) Italian Multicenter Study. This study included 8260 Caucasian type 2 diabetes patients from nine centers from whom three to five A1C readings were obtained over a 2-year period. Mean A1C and A1C-SD was 7.57% and 0.46%, respectively. The variability in A1C was independently correlated with albuminuria and albuminuric CKD, but not with non-albuminuric CKD [25].

There are several possible mechanisms to explain the association between A1C variability and nephropathy. First, increased A1C variability could be a signal of previous poor glycemic control. The metabolic memory phenomenon suggests that diabetic nephropathy continues to occur even after good control has been established [26]–[27]. Second, highly variable A1C was associated with some baseline factors, such as increased smoking, higher blood pressure, and an increased prevalence of peripheral neuropathy and peripheral vascular disease [6], [13], [17]–[18]. Therefore, A1C variability might be indicative of unhealthy lifestyle behavior, non-compliance, comorbidities, or complications associated with the development of diabetic nephropathy. Finally, a number of primary studies revealed that glucose fluctuations might activate oxidative stress, which plays a major role in the development of diabetic complications [28]. However, most studies were based on short-term glycemic variability [29]–[30]. Therefore, future studies should assess whether long-term glycemic variability affects oxidative stress.

To the best of our knowledge, this study is the first meta-analysis to assess the association between A1C variability and the risk of progression of renal status. The eight included articles were either cohort or post-RCT studies, mostly with a NOS score of ≥7; therefore, they improved the quality of the final results. Moreover, sufficient subgroup and sensitivity analyses contributed to the stability of this review.

However, some limitations should also be noted. First, among the included studies differences in A1C measurements, the frequency in testing, the interval time between tests, and the methods used to calculate SDs all contribute to the heterogeneity of the data. Although the results of sensitivity analysis were stable, a uniform and standard measurement should be established for A1C variability in the future. Second, the definition for the renal end-point varies among studies, although the results based on a combined definition were significant. Further studies should be performed to specify the relationship between A1C variability and different kinds of renal outcome. Third, although confounding factors were partially adjusted using multivariate regression analysis in most of the included studies, this remains a limitation because of the failure to control all factors simultaneously in observational studies. Further studies should assess the effect of reducing A1C variation on nephropathy complications. Finally, the result of Egger's test, which might be doubtful when the sample size is <20, suggested a potential publication bias in this review. Therefore, trim and fill analyses were performed and missing studies had no significant influence on the results.

In conclusion, high A1C variability, a parameter of long-term glycemic variability, increases the risk of nephropathy. These results suggest that A1C variability is an important component of the primary and secondary prevention of diabetic complications. Meanwhile, developing a standardized system to evaluate A1C variability and perform interventional studies is critical to assess the correlation between therapies that reduce A1C variability and renal prognosis. Moreover, additional studies should explore the physiopathological mechanism of A1C instability that leads to renal complications.

## Supporting Information

S1 Checklist **PRISMA Checklist.**
(DOC)

S1 File **Search strategy.**
(DOC)
