# Supplementary material for: HbA1C Variability and the Risk of Renal Status Progression in Diabetes Mellitus: A Meta-Analysis
Source: PLoS One. 2014 Dec 18;9(12):e115509. doi: 10.1371/journal.pone.0115509 (PMC4270779; doi:10.1371/journal.pone.0115509)
Supplement: S1 File — Search strategy. (DOC) [file pone.0115509.s002.doc]

**Pubmed**

#1 Hemoglobin A, Glycosylated [MESH]

#2 HbA1c [TW]

#3 A1c [TW]

#4 glycated hemoglobin [TW]

#5 variation [TW]

#6 variability [TW]

#7 (#1 OR #2 OR #3 OR #4) AND (#5 OR #6)

#8 HbA(1c) variability [TW]

#9 HbA(1c) variation [TW]

#10 #7 OR #8 OR #9

#11 Albuminuria [mesh]

#12 Proteinuria [mesh]

#13 Diabetic Nephropathies [mesh]

#14 "Renal Insufficiency, Chronic"[Mesh]

#15 Albuminuria [TW]

#16 Proteinuria [TW]

#17 urinary albumin excretion [TW]

#18 microalbuminuria [TW]

#19 nephropathy [TW]

#20 microvascular complications [TW]

#21 chronic kidney disease* [TW]

#22 #11 OR #12 OR #13 OR #14 OR #15 OR #16 OR #17 OR #18 OR #19 OR #20 OR #21

#23 #10 AND #22

**Embase**

#1 exp glycated hemoglobin/

#2 HbA1c.tw

#3 A1c.tw

#4 glycated hemoglobin.tw

#5 variation .tw

#6 variability.tw

#7 (#1 OR #2 OR #3 OR #4) AND (#5 OR #6)

#8 exp Albuminuria/

#9 exp Proteinuria/

#10 exp diabetic nephropathy/

#11 exp chronic kidney failure/

#12 Albuminuria [TW]

#13 Proteinuria [TW]

#14 urinary albumin excretion [TW]

#15 microalbuminuria [TW]

#16 nephropathy [TW]

#17 microvascular complications [TW]

#18 chronic kidney disease$.tw.

#19 #8 OR #9 OR #10 OR #11 OR #12 OR #13 OR #14 OR #15 OR #16 OR #17 OR #18

#20 #7 OR #19
